# Experimental Study on the Durability of Steel Anchors for Prestressed CFRP Laminates under Accelerated Galvanostatic Corrosion

**DOI:** 10.3390/ma15165665

**Published:** 2022-08-18

**Authors:** Jun Deng, Minting Zhong, Yifeng Zheng, Miaochang Zhu

**Affiliations:** 1 School of Civil Engineering, Guangzhou University, Guangzhou 510006, China; 2 School of Civil and Transportation Engineering, Guangdong University of Technology, Guangzhou 510006, China

**Keywords:** anchors, accelerated corrosion, durability, anchor efficiency

## Abstract

The novelty of the present study is to address the durability of corroded anchors for prestressing CFRP laminates. Two types of steel anchors, clamp anchors and wedge anchors, were used to prestress CFRP laminates and then subjected to steel corrosion through a galvanostatic acceleration approach, which was followed by tensile tests. Compared to clamp anchors, wedge anchors showed a superior durability performance in terms of their prestress retention, anchor efficiency, and resistance to the slippage of the CFRP laminate. After accelerated corrosion for 144 h, the clamp anchor exhibited a prestress retention of 79.1% and an anchorage efficiency of 55%, and the percentages became 9.0% and 100% for the wedge anchor. The slippage rates of the clamp anchor and the wedge anchor were 0.036 mm/kN and 0.026 mm/kN, respectively. Therefore, the wedge anchor, which exhibited higher prestress tension and anchorage efficiency, performed better than the clamp anchor. The present work provides an apparatus for exploring the corrosion-induced durability of steel anchors and experimental evidence that helps refine the provision in the guidelines for addressing anchor durability.

## 1. Introduction

Carbon fiber-reinforced polymers (CFRPs) have turned out to be a popular alternative for the new construction, rehabilitation, retrofitting, and strengthening of aging steel and concrete structures [1,2]. The application of CFRPs can be used to enhance the structural performance and extend the service life; therefore, the demand for new construction for production and living can be reduced. Reducing construction results in a pronounced drop in CO_2_ emissions and energy consumption. Thus, utilizing CFRPs can be seen as a prospective approach of significant contribution to the sustainability of construction industries.

As one of the typical application scenarios, CFRPs can be externally attached to ageing structures via adhesive bonding. Various investigations have shown that upgraded structures fail in different patterns, of which debonding of the CFRP (also called detachment) is the primary failure mode [3,4,5,6,7]. Debonding failure can be controlled by limiting the interfacial bond stress, which results in the CFRP tensile stress being fairly lower than its tensile capacity [8]. Another issue is associated with the adverse effects of environmental attacks, leading to a further decrease in the CFRP stress [9,10,11]. Hence, debonding failure and adverse environmental effects prevent the full-strength exploitation of CFRPs, constituting a major barrier to their wider application in construction industries.

For CFRPs to achieve a higher utilization efficiency, prestressing techniques have been developed, which have turned out to be green, safe, and reliable. The use of prestressed CFRPs can effectively increase structural performance, such as the flexural performance of bending members [12,13,14,15,16,17,18]. The resistance to fatigue of the strengthened structures with prestressed CFRPs can be enhanced as well [19,20,21,22]. Applying reliable anchors to maintain the stress in the prestressed CFRPs is a requisite for increasing the structural performance and obtaining a higher utilization of CFRPs [23]. Therefore, much attention has been directed to developing anchors for gripping CFRPs.

Two categories of anchors have been widely studied: epoxy-based anchors and mechanical anchors [24]. Previous studies have focused on anchor configurations, gripping mechanisms, anchor efficiency, and geometrical optimization [15,25,26,27,28,29,30,31,32,33,34]. According to a literature review [24], epoxy-based anchors show a lower anchor efficiency and a long and complicated installation process, especially as they require sufficient curing time of the adhesive, while mechanical anchors can reach an efficiency up to the CFRP tensile strength and just require simple installation. It was also pointed out that the anchors developed for a particular size of a CFRP cannot be extrapolated to CFRPs with different sizes; therefore, the development of anchors represents a continuing motivation for the prestressing CFRP technique. In addition, mechanical anchors are usually made of steel and susceptible to corrosion issues, especially in many servicing conditions of the ageing structures, such as infrastructures in coastal and offshore regions. However, the effects of steel corrosion on the durability of mechanical anchors remain unclear.

The research novelty is to address the long-term performance of anchors suffering from corrosion, providing durability data for evaluating or choosing adequate anchors for the prestressing CFRP technique. Two commonly used types of anchors, clamp anchors and wedge anchors, were investigated in the present study. The anchors were pretensioned and then subjected to accelerated corrosion. Accelerated corrosion was accomplished by a galvanostatic approach involving connecting the anchors to the positive terminal of the constant current source. The stress loss in the pretensioned CFRP was monitored using Fiber Brag Grafting (FBG) during accelerated corrosion. Tensile tests were performed to examine the corrosion effects on the anchors in terms of the anchor efficiency and resistance to CFRP slippage.

## 2. Experimental Program

### 2.1. CFRP and Anchor Details

**Figure 1:**
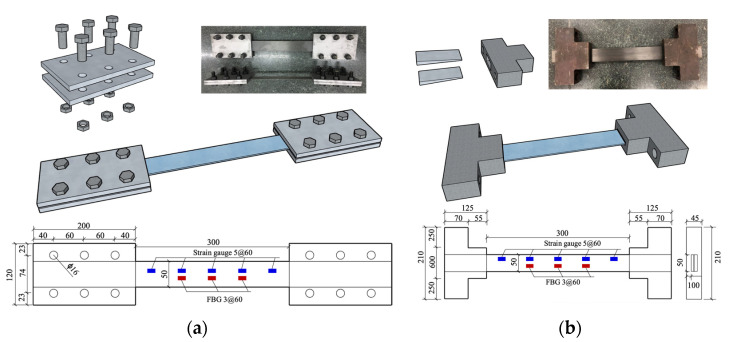
Clamp anchor (**a**) and wedge anchor (**b**).

The CFRP used in this study was precured laminates with a cross-section of 50 mm × 1.4 mm. The ultimate tensile strength of the CFRP laminates was 2600 MPa. A total of 12 anchors, 6 clamp anchors and 6 wedge anchors, were studied. All the anchors were carefully selected so that the CFRP laminates could be sufficiently gripped and fail before the anchors. The clamp anchors consisted of two steel plates threaded using 6 high-strength M8.8 bolts 16 mm in diameter, as shown in Figure 1a. The steel plates were made of Q235 steel and had dimensions of 200 mm × 120 mm × 10 mm. Bolt holes spaced at 60 mm were punched in two lines. The steel plate surface in contact with CFRP laminates was sandblasted to increase the friction.

The wedge anchors were manufactured and provided by Nanjing Haituo Composite Material Co., Ltd. (Nanjing, China) The wedge anchors, composed of two wedges and a barrel, are shown in Figure 1b. The barrel was machined from a 45# steel prism, from which a wedge hole was formed using a precise line cutting method. The barrel’s cross-section was 90 mm × 45 mm at the loaded end and 210 mm × 45 mm at the free end.

### 2.2. Pretensioning of CFRP Gripped by Anchors

Six CFRP laminates were used in the study, three gripped by clamp anchors and three gripped by wedge anchors. For clamp anchors, the sandblasted surface of the steel plates was cleaned using acetone and dried in air, and CFRP laminates were then bonded to the sandblasted surface. A two-component epoxy resin with a base-to-hardener mixing ratio of 2:1 in weight was used as the adhesive, in which 1 mm diameter ballotinis in 1 wt% of the adhesive were blended to control the adhesive thickness. The adhesive was applied onto the contact surface between the CFRP laminate and steel plates, and the CFRP laminate was therefore sandwiched between the two steel plates. Note that the longitudinal centerline of the CFRP laminate and the anchor should overlap. The sandwiched CFRP laminates were placed in the laboratory until the adhesive was sufficiently cured. The clamp anchors were then tightened using 6 bolts. A torque wrench was used to impose a torque of 100 N·m on each bolt, and the resulting tightening force was 45.5 kN, which did not exceed the bolt tightening limit of 80 kN.

As for the wedge anchors, CFRP laminates were gripped following a two-step procedure. The CFRP laminate placed between the two wedges was threaded through the wedge cut in the barrel. Once the two wedges were seated, the CFRP laminate was tensioned away from the loaded end to achieve a firm contact between the wedges and the barrel. Subsequently, the tensioning force of the CFRP laminate was released, and the wedges tended to slide away from the wedge cut due to the recovery of elastic deformation. The sliding trend of the wedges can be prevented because of the presence of friction force between the wedges and barrel, providing the effective self-locking of the wedges.

**Figure 2:**
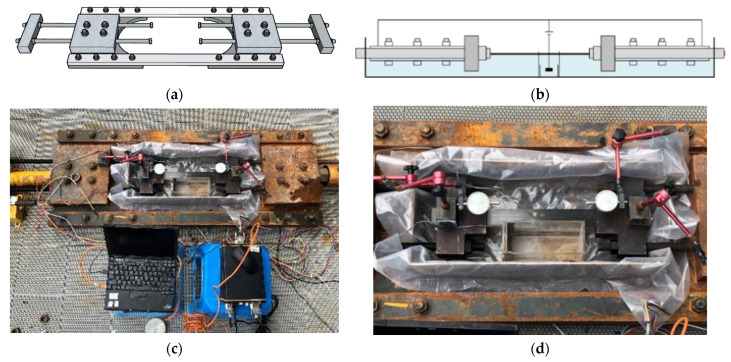
Test setup for pretensioning and accelerated corrosion: (**a**) frame for pretensioning, (**b**) electric circuit, (**c**) overview, and (**d**) close-up view for acceleration corrosion.

Figure 2 shows the exclusively made apparatus for prestressing the anchored CFRP laminates. The anchors used to grip the CFRP laminates were connected to the baffles through the bearing plates using two bolts. A loading cell was installed between the bearing plate and the baffle at one end of the apparatus to measure the pretension force. A jack placed at the other end was employed to apply pretensioning. The anchored CFRP laminates were pretensioned at a designed stress level equal to 20% of the tensile capacity. To compensate for the stress loss caused by release, 5% overtension was applied. Once the pretensioning was completed, lock nuts were used to hold the bolts connecting the bearing plate and the baffle.

### 2.3. Accelerated Galvanostatic Corrosion

Steel anchors tend to corrode in natural environments, especially in hot and humid conditions; therefore, there is a need to investigate the effect of steel corrosion on the durability of anchors. As the corrosion kinetics are naturally slow for anchors used in realistic conditions, accelerated galvanostatic corrosion was adopted to introduce significant amounts of corrosion to the anchors within a shortened period. This galvanostatic corrosion approach has also been used to investigate the long-term performance of reinforcing steel in concrete [35] and CFRP-steel joints [36]. The advantage of galvanostatic corrosion lies in its capacity to control the corrosion to an expected degree by adjusting the energization current or voltage and duration.

After pretensioning the CFRP laminates, the anchors were subjected to accelerated galvanostatic corrosion by connecting them to the positive terminals of the constant current suppliers. Therefore, a self-devised apparatus for simultaneously applying tensioning and accelerated corrosion to the anchors was proposed and patented. The apparatus can be used to monitor the prestress and slippage of the anchored CFRP laminates when equipped with adequate instruments. The apparatus for implementing galvanostatic corrosion is presented in Figure 2. The anchors at both ends of the prestressed CFRP laminates were connected in parallel to the positive terminals of the constant current suppliers, and an inert cathode was surrounded by a cation exchange membrane connected to the negative terminals. The arrangement of two parallel circuits alleviated the electricity-induced damage to the CFRP laminate. The cation exchange membrane prevented the passage of hydroxide ions produced at the cathode to the saline electrolyte. The anchors were half-immersed in the 3.5% saline solution that functioned as an electrolyte, while the CFRP laminate was kept above the solution level; therefore, the damage of the laminate caused by the imposed current could be avoided [37]. The energization current was 2.5 mA, and two durations of 72 and 144 h were considered. The expected weight loss in the anchors was 375.12 g after 144 h of energization according to Faraday’s first law, corresponding to 10.72 wt% of the clamp anchor and 5.77 wt% of the wedge anchor. According to the anchor type and energization duration, the anchors were designated in the form of C/W-y, where C or W indicates the clamp anchor or the wedge anchor, respectively, and y represents the energization duration. For instance, C-144 indicates the clamp anchor subjected to accelerated corrosion for 144 h.

**Figure 3:**
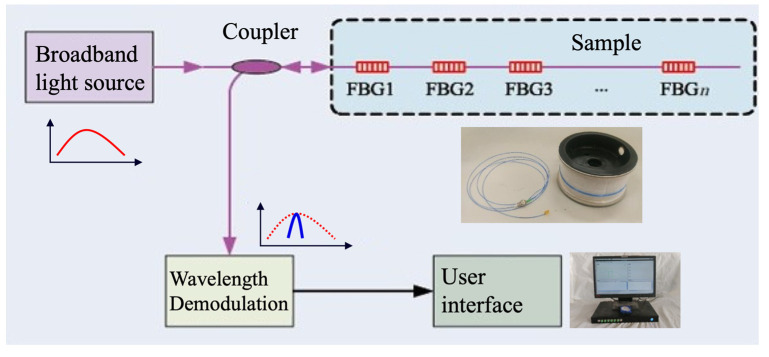
FBG for strain monitoring.

Applying FBG has proven to be a reliable technique for strain monitoring, especially for obtaining strain distribution, as shown in Figure 3. In the present study, the strain evolution of prestressed CFRP laminates during accelerated corrosion was monitored using FBG. The FBG sensor was attached along the longitudinal centerline of the CFRP laminates, and the strains at three locations were monitored and recorded, as shown in Figure 1.

As the wavelength of FBG can be affected by both the stress and conditioning temperature, it is necessary to separate the contribution of the CFRP strain. It has been found that the FBG wavelength is proportional to strain and temperature, as expressed in Equation (1).

(1) $$\frac{\Delta \lambda_{B}}{\lambda_{B0}}=c_{\varepsilon }\varepsilon +c_{T}\Delta T$$

where ***λ_B_***_0_ is the original wavelength of FBG, Δ***λ_B_*** is the total shift in the FBG wavelength caused by strain and temperature, and ***c_ε_*** and ***c_T_*** are the coefficients considering the effects of strain and temperature, respectively. ε is the strain and ΔT is the temperature change. The value of ***c_ε_*** can be taken as 0.783 × 10^−6^ ***με***^−1^ [38], while a value of 3 × 10^−6^ °C^−1^ was assigned to ***c_T_*** according to field calibration. The temperature variation was measured using a thermometer during accelerated corrosion. An interrogator was utilized to demodulate the collected light signals to obtain strain data.

### 2.4. Uniaxial Tension Tests

After completing accelerated corrosion, uniaxial tensile tests were performed to examine the effects of steel corrosion on the anchor efficiency and CFRP slippage. The tension force was applied using the same apparatus for CFRP pretensioning. The CFRP laminates were loaded in a stepwise procedure. An increment of 10 kN was applied until 50 kN was reached, and the applied force at each step was maintained for 1 min. After that, the force increment was reduced to 5 kN, and the holding time changed to 0.5 min. Five electrical strain gauges were mounted on the CFRP surface to measure the strain during tests. A linear variable differential transducer (LVDT) was used to measure the slippage of the CFRP laminates out of the anchors.

### 2.5. Long-Term Prestress Loss Monitoring

The durability performance of the two types of anchors can be evaluated and compared by conducting the above experiments. The anchor exhibiting better performance was further tested in terms of prestress retention during an extended period of accelerated corrosion. The apparatus for applying the pretensioning and accelerated corrosion was the same as shown in Figure 2. The prestress level is also taken as 20% of the tensile capacity of the CFRP laminate. Besides, the same energization current of 2.5 mA was imposed, while the energization time was extended up to 400 h. The tensile strain of the anchored CFRP laminate by means of FBG was monitored during accelerated corrosion, as shown in Figure 1.

## 3. Test Results and Discussion

### 3.1. Accelerated Corrosion and Prestress Loss Monitoring

**Figure 4:**
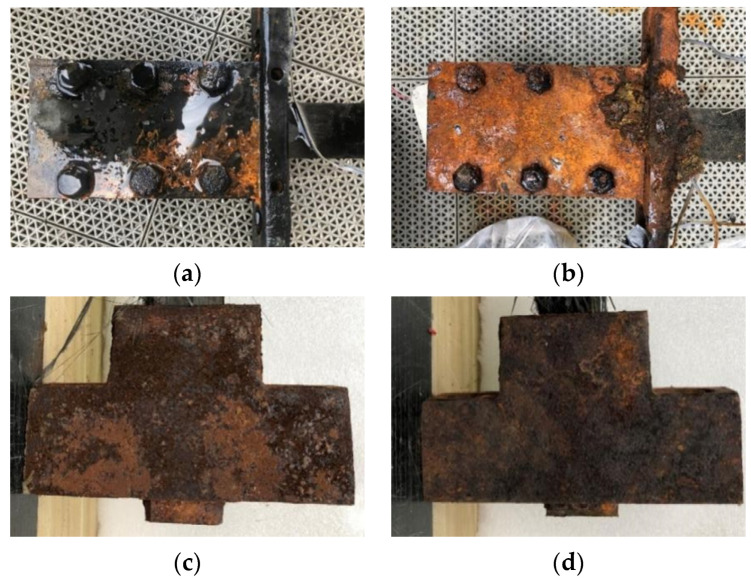
Clamp anchors and wedge anchors after accelerated corrosion. (**a**) C-72. (**b**) C-144. (**c**) W-72. (**d**) W-144.

The appearance of the anchors after accelerated corrosion was inspected. Figure 4 shows the anchors subjected to accelerated corrosion. For Anchor C-72, corrosion was mostly concentrated on the steel plate near the bolts, and minor corrosion was observed on the bolts. For Anchor C-144, significant corrosion occurred on both the steel plate and bolts, and the outer rust layer was delaminated from the steel substrate. The corrosion products were spread around the whole anchor for the corroded wedge anchors, and a longer energization duration led to more significant corrosion.

**Figure 5:**
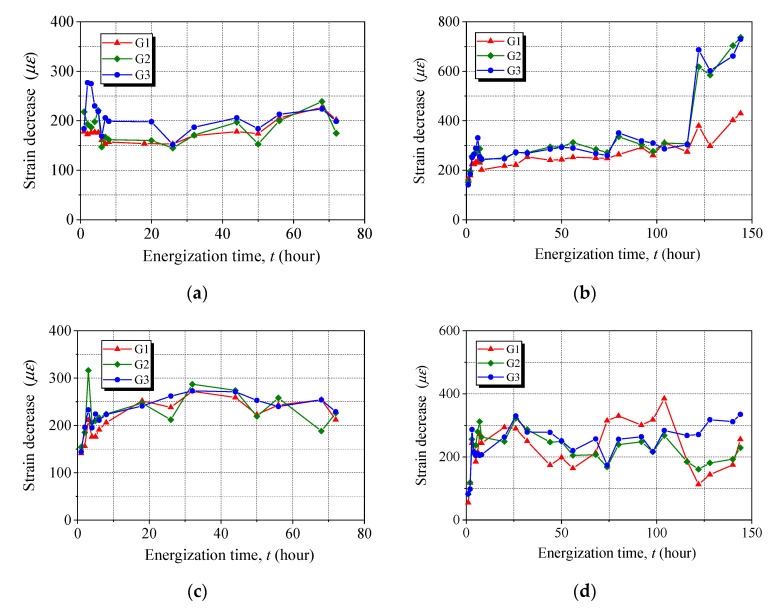
Strain evolution of anchored CFRP laminates during accelerated corrosion. (**a**) C-72. (**b**) C-144. (**c**) W-72. (**d**) W-144.

Figure 5 presents the strain evolution of the anchored CFRP laminates during accelerated corrosion. The strain values recorded at three locations were close, except for one strain measurement for Anchor C-144, which was due to the separation of the FBG from the CFRP laminate, and the results were abandoned in calculating the average strain. All the anchors exhibited an early decrease immediately after implementing energization and then a plateau until the end of accelerated corrosion, except for Anchor C-144, for which a significant decrease was observed at 122 h. The early decrease in the prestress loss was observed (see Figure 5), and we believe such prestress loss is caused by significant steel corrosion that can occur around the anchors after a few hours of energization. With an increase in energization time, most of the corrosion products were localized on the anchors’ surface in contact with the electrolyte, and as a result, a gentle effect on the prestress loss could be observed, indicated by the plateau. The proposed apparatus was capable of maintaining the applied prestress to the anchors subjected to accelerated corrosion.

**Figure 6:**
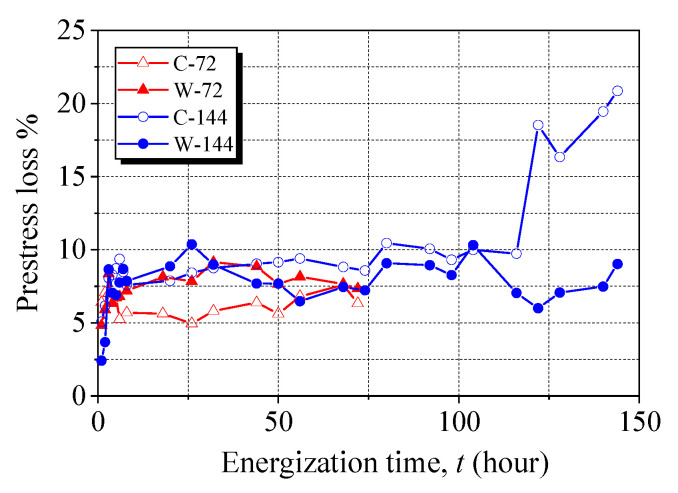
Prestress loss of anchored CFRP laminates during accelerated corrosion.

To evaluate the capacity of maintaining prestress, the average strain of the anchored CFRP laminates was used to calculate the stress, which is compared in Figure 6. In the initial 8 h of energization, the prestress losses were 5.7% and 7.6% for Anchors C-72 and C-144, respectively, while the respective percentages were 7.2% and 7.9% for Anchors W-72 and W-144. The slight discrepancy in the initial prestress loss observed for the anchors was due to differences in anchor manufacturing, anchor geometries, bolt tightening, wedge seating, and pretensioning implementation. At the end of the 72-h energization, little difference in the prestress loss of the anchors was noticed. With increasing energization time to 122 h, the prestress level was gradually reduced for Anchor C-144, and the prestress loss was 20.9% after 144 h. The significant prestress loss in the corroded clamp anchors was mainly associated with the severely corroded bolts, which led to a reduced tightening force of the bolts. Thus, the friction force between the CFRP laminate and the steel plate was decreased. The wedge anchor showed a superior advantage in maintaining the prestress, as only a 9.0% stress loss was observed after 144 h of energization. This is due to the expansive corrosion products being entrapped inside the barrel, maintaining adequate friction between the wedges and barrel. Compared to Anchors W-72 and W-144, an additional 72 h of energization resulted in a minor change in the prestress loss, indicating good resistance of the wedge anchors to steel corrosion.

### 3.2. Tensile Tests

#### 3.2.1. Failure of CFRP Laminates

**Figure 7:**
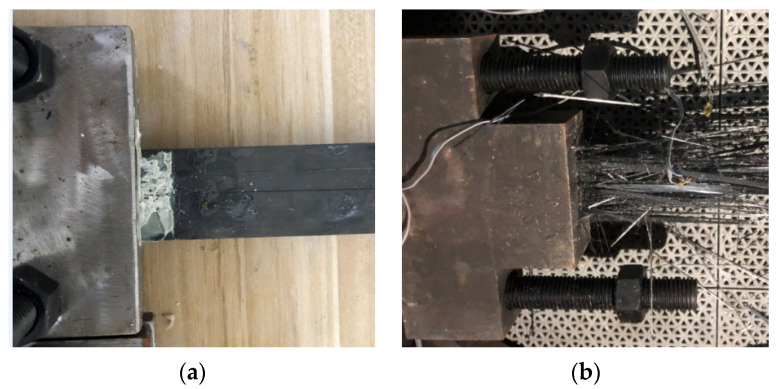
Failure modes of anchored CFRP laminates. (**a**) Splitting failure. (**b**) Tensile fracture.

Different failure modes of the anchored CFRP laminates were observed after tensile tests, depending on the type of anchors. Figure 7 presents the failure modes of the anchored CFRP laminates. The CFRP laminates with noncorroded or corroded clamp anchors exhibited splitting failure featuring a longitudinal through crack along the laminate centerline. This is because the contact pressure normal to the CFRP laminate is minimal at the centerline, which has the farthest distance from the tightened bolts. Therefore, an uneven stress distribution occurred across the section of the CFRP laminates, facilitating the occurrence of splitting. Once splitting failure occurs, the ultimate tensile strength of the CFRP laminates cannot be achieved. For the wedge anchors, the CFRP laminates fractured near the loaded end of the anchors, and a higher ultimate applied force can be anticipated, indicating a superior anchorage performance.

#### 3.2.2. Tensile Response of Anchored CFRP Laminates

**Figure 8:**
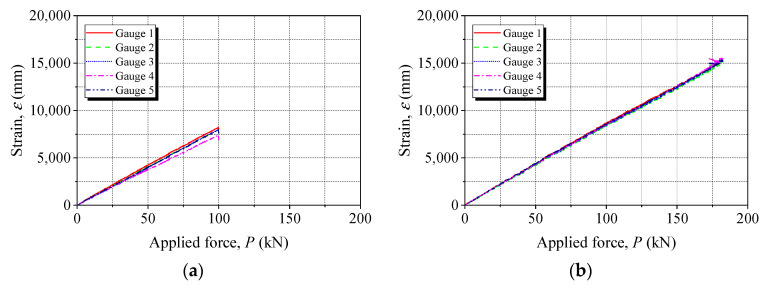
Tensile response curves of anchored CFRP laminates after 144-h energization. (**a**) C-144. (**b**) W-144.

**Figure 9:**
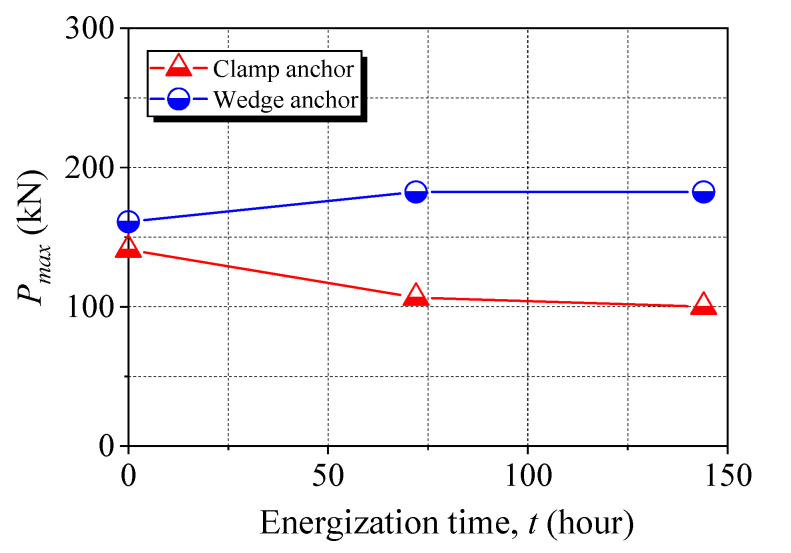
The ultimate applied force obtained from tensile tests.

Figure 8 shows the tensile response curves of the anchored CFRP laminates after 144 h of energization. There were five strain gauges attached to each anchored CFRP laminate, as indicated in the legend of Figure 8, and the corresponding tensile stress–strain curves can be plotted. All the CFRP laminates showed a linear tensile behavior. No significant difference was observed in the five tensile curves for each anchored CFRP laminate. The ultimate applied force (*P_u_*) of the CFRP laminates is presented in Figure 9. Compared with the noncorroded anchor (C-0), *P_u_* of the CFRP laminates gripped with Anchors C-72 and C-144 decreased by 24% and 29%, respectively, while a 13% gain can be seen for both Anchors W-72 and W-144. The effects of steel corrosion on different anchors were associated with the failure pattern of the anchored CFRP laminates, which has been interpreted. This indicates the superior durability performance of the wedge anchor in resisting steel corrosion-induced degradation.

### 3.3. Anchor Efficiency

**Figure 10:**
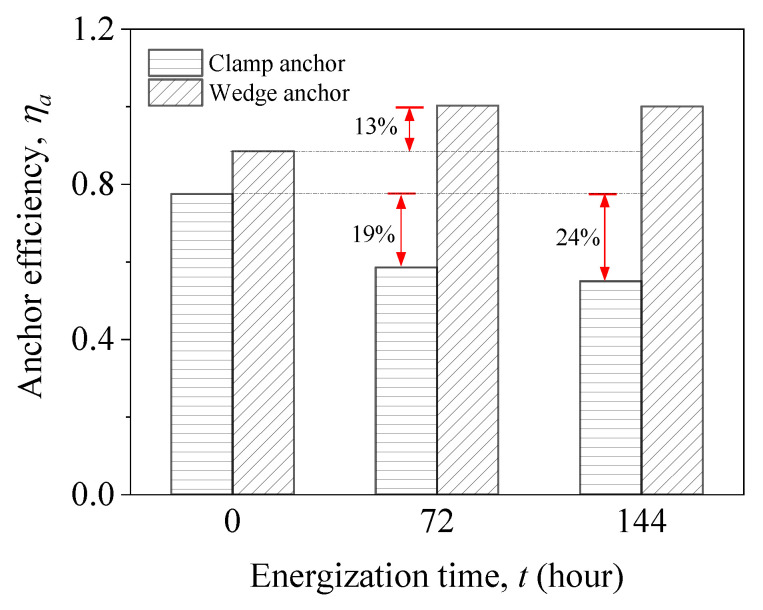
Changes in the anchor efficiency of anchors after accelerated corrosion.

Anchor efficiency is of great importance for the development and optimization of reliable anchors for prestressed CFRP strengthening, which is vital in the evaluation of the long-term durability performance of the anchor. The anchor efficiency (*η_a_*) is defined as the ratio of the maximum applied stress of the anchored CFRP laminate to its tensile strength. Figure 10 shows the anchor efficiency of anchors after accelerated corrosion. For noncorroded anchors, *η_a_* of the wedge anchor is 89%, which is 11% higher than that of the clamp anchor. The ultimate force that can be carried by the epoxy-based anchors normally amounts to a small fraction (36–60%) of the CFRP tensile capacity [24]. Therefore, the non-corroded clamp and wedge anchors have a more satisfactory performance in the anchor efficiency.

As the energization time increases, a decrease in *η_a_* is noticed for clamp anchors, while wedge anchors exhibit the opposite trend. After 72 h of accelerated corrosion, *η_a_* of the clamp anchor was reduced by 19%, and a total loss of 23% in *η_a_* was found with additional 72-h corrosion. The *η_a_* loss for the clamp anchors was mainly attributed to the reduced tightening force of the corroded bolts. For wedge anchors, accelerated corrosion leads to *η_a_* increasing up to 100%, indicating fulfillment of the tensile strength of CFRP laminates. This is due to the expansive corrosion products being entrapped inside the wedge anchors, and the friction at the barrel-wedge interface was enhanced. The good anchorage durability renders the wedge anchors more adequate for practical use in prestressed CFRP strengthening, especially in the case of severe steel corrosion.

### 3.4. Slippage of Anchored CFRP Laminates

The effects of accelerated corrosion on the anchor resistance to the slippage of gripped CFRP laminates were evaluated. Since the displacement measured using the LVTD includes the CFRP and bolt elongation, excluding the CFRP and bolt deformation is necessary when evaluating the slippage. The CFRP elongation can be calculated using the average strain obtained from five gauges, and the bolt elongation can be evaluated using the measured applied force. The equation for calculating the slippage (*δ*) of the gripped CFRP laminate is expressed as follows Equation (2).

(2) $$\delta =u-\varepsilon L_{0}-\frac{P}{2E_{s}A_{s}}L_{s}$$

where *u* is the displacement measured by the LVDT; *ε* is the CFRP strain taken as an average of measurements from five strain gauges; *L*_0_ is the length of the CFRP laminates between the loaded ends of the anchors; *P* is the measured applied force; *E_s_* is Young’s modulus of the steel; and *A_s_* and *L_s_* are the cross-sectional area and the length of the bolts connecting the anchor to the baffle, respectively.

**Figure 11:**
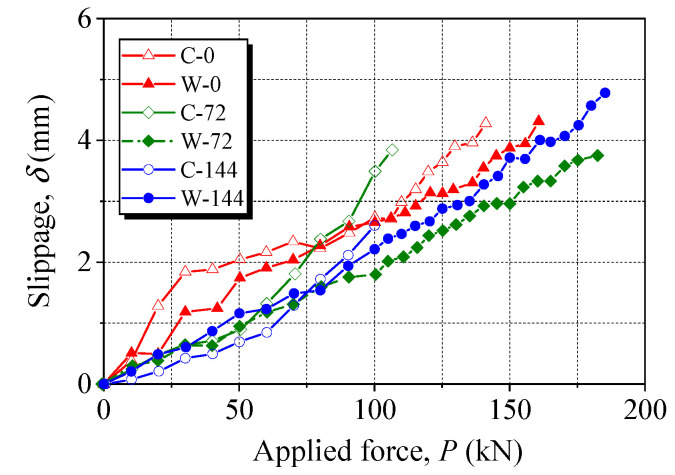
Slippage of anchored CFRP laminates during tensile tests.

Figure 11 presents the slippage of anchored CFRP laminates during tensile tests. With an increase in the applied force, *δ* exhibits a monotonic increasing trend up to CFRP failure. At the same applied force, *δ* is generally higher for the clamp anchors than wedge anchors. For clamp anchors, *δ* for the noncorroded anchor increased faster than for the corroded anchors up to 60 kN and slower with a further increase in the applied force. For wedge anchors, *δ* represents an almost linear behavior as the applied force is increased, and the CFRP laminates gripped by the corroded anchors have a smaller *δ* than the CFRP laminate gripped by the noncorroded anchor. The values of *δ* at failure for the clamp anchor and the wedge anchor after 144 h of energization are 3.843 mm and 4.781 mm, respectively, because the wedge anchor carried a much higher maximum applied force. To quantify the resistance of different anchors, the slippage rate, defined as the ratio of the slippage to the applied force at failure, was calculated. The slippage rates of the clamp anchor and the wedge anchor after 14 h of energization are 0.036 mm/kN and 0.026 mm/kN, respectively. This indicates that the wedge anchors perform better in resisting the slippage of the gripped CFRP laminates.

### 3.5. Long-Term Prestress Loss of the CFRP Laminate with a Wedge Anchor

**Figure 12:**
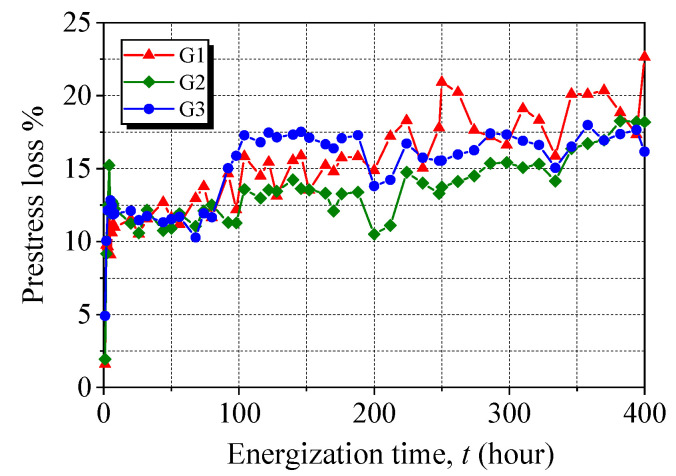
Prestress loss of the CFRP laminate with a wedge anchor during 400-h accelerated corrosion.

Based on the above experimental results and analyses, wedge anchors performed better than clamp anchors in terms of the prestress retention, anchor efficiency, and resistance to the slippage of the CFRP laminate. Thus, the wedge anchor was further tested during extended accelerated corrosion for up to 400 h. Figure 12 presents the prestress loss of the CFRP laminates anchored with a wedge anchor after 400 h of accelerated corrosion. Significant prestress loss is found at the early stage of accelerated corrosion, which is consistent with previous findings. A slow increase in prestress loss then occurs with increasing energization time. This is because more corrosion products can be observed in the barrel and wedge of the wedge anchor within the extended accelerated corrosion. The 400-h accelerated corrosion resulted in a 19% prestress loss, which is approximately two times higher than that observed for the CFRP laminate with the 144-h corroded wedge anchor. According to Faraday’s law, the degree of accelerated corrosion, if evaluated based on the weight loss of anchors, is 2.8 times higher for the 400-h corroded wedge anchor than the 144-h counterpart. Therefore, the increasing rate of prestress loss is reduced with increasing energization time, indicating that the wedge can also perform well in maintaining prestress during the extended accelerated corrosion.

## 4. Conclusions

Two types of mechanical anchors, clamp anchors and wedge anchors, were investigated in terms of their prestress loss, anchor efficiency, and resistance to slippage of the CFRP laminates under accelerated corrosion. The anchorage systems consisting of the anchors and CFRP laminates were pretensioned and then subjected to steel corrosion through a galvanostatic acceleration approach. Afterwards, uniaxial tensile tests were performed on the anchorage systems. Based on the experimental results, the following conclusions can be drawn in terms of prestress retention, anchorage efficiency, and resistance to slippage of the CFRP laminate.

The prestress loss of the anchored CFRP laminates was evaluated by measuring the CFRP strain. The results show that the prestress loss was 20.9% and 9.0% for the clamp anchor and wedge anchor, respectively, after accelerated corrosion for 144 h, indicating that the wedge anchor showed a better prestress retention under corrosion. When accelerated corrosion extended up to 400 h, the wedge anchor also performed well with a reduced increasing rate of the prestress loss.

The CFRP laminates featured a splitting pattern for the clamp anchors and tensile fracture for the wedge anchors, irrespective of accelerated corrosion. The wedge anchors had a higher ultimate applied force than the clamp anchors.

The anchor efficiencies were 78% and 89% for the clamp anchor and the wedge anchor without accelerated corrosion, respectively, while the percentages become 55% and 100% after 144 h of energization. Even after enduring severe corrosion, the wedge anchor’s anchor efficiency increased to a level that could guarantee the CFRP tensile failure, indicating that the wedge anchors can grip the CFRP laminates efficiently, even under severe corrosion.

The slippage at failure for the clamp anchor and the wedge anchor after 144 h of energization was 3.843 mm and 4.781 mm, respectively. The larger slippage endured by the wedge anchor was due to the wedge anchor carring a much higher maximum applied force. The slippage rates of the clamp anchor and the wedge anchor after 144 h of energization were 0.036 mm/kN and 0.026 mm/kN, respectively, indicating that the wedge anchors exhibited better resistance to the CFRP slippage.

Therefore, it can be concluded that wedge anchors are superior to clamp anchors in terms of their prestress retention, anchor efficiency, and resistance to CFRP slippage. The self-devised and patented apparatus has been shown to be adequate for exploring the corrosion-induced durability of steel anchors for prestressing CFRP laminates, contributing to the development of standardized test methods. The experimental results also provide solid evidence for the use of wedge anchors when designing a durable anchorage system, which helps refine the relevant provisions in the guidelines for addressing anchor durability.

## Data Availability

Not applicable.
